# Firing Hot, Firing Cold: How Poikilotherms Compensate for Temperature Swings

**DOI:** 10.1371/journal.pbio.1000470

**Published:** 2010-08-31

**Authors:** Richard Robinson

**Affiliations:** Freelance Science Writer, Sherborn, Massachusetts, United States of America

**Figure pbio-1000470-g001:**
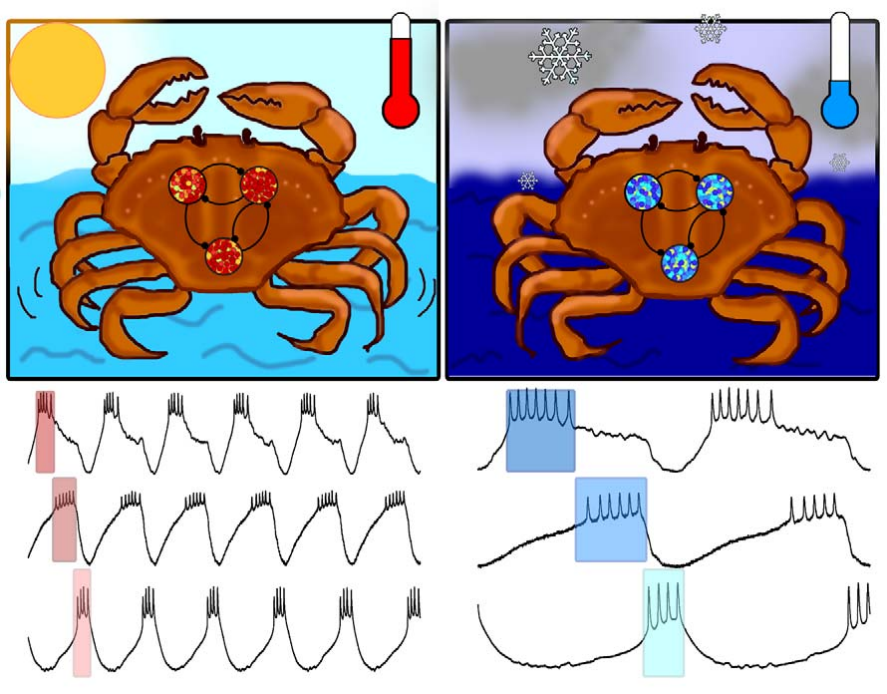
Crabs in warm conditions exhibit faster network frequencies than crabs in cold conditions. However, the motor pattern remains constant in both conditions while network parameters manifest disparate temperature dependencies. (Image: Gabrielle Gutierrez) .

[Fig pbio-1000470-g001]If you are reading this, you have one less problem than the crabs that are the subject of a new study in this issue of *PLoS Biology*. Humans, like all mammals (as well as birds), are “warm-blooded,” or more correctly “homeothermic,” meaning we maintain our core body organs at a constant temperature. We expend considerable energy to do so, and may die when we cannot. In contrast, crabs, cod, caterpillars, and the like are “cold-blooded,” or “poikilothermic,” with body temperatures that change with the environment they are in. When the mercury plunges, a poikilotherm, and everything that happens within it, merely slows down.

At the most basic level, all those things happening within the poikilotherm are chemical reactions, and it is their precise coordination that allows the organism to keep on living. But chemists have long known that reactions differ in the degree to which they will speed up and slow down in response to temperature changes. So here is the problem the crab has that we do not: how does it make sure that processes relevant to a given behavior remain coordinated across a wide range of temperatures?

Lamont Tang, Eve Marder, and colleagues set out to answer this question for a group of neurons in *Cancer borealis*, a North Atlantic crab that flourishes in intertidal and subtidal ecosystems where the water temperature may swing as much as 15°C in a day and more than 20°C between winter and summer. Chewing and filtering of food is driven by a rhythmic motor pattern maintained by the coordinated firing of a small network of neurons. The neurons fire in a specific sequence with time lags between them. To maintain effective chewing and filtering, those temporal relationships should remain the same—that is, they must remain “in phase” with each other, even if the overall frequency of firing changes with temperature.

The authors began by confirming that this was so, showing that while the firing frequency increased almost four-fold between 7°C and 23°C, the phase relationships were unaltered. In the language of the physical chemist, a system's responsiveness to a temperature change is known as its “Q_10_”. A reaction that doubles its rate with a 10-degree rise in temperature has a Q_10_ of 2, while one that changes not at all has a Q_10_ of 1. Thus, while the frequency had a Q_10_ of 2.3, the phase relationships had a Q_10_ of 1, displaying almost perfect “temperature compensation.”

They next asked what features of the system might account for this temperature compensation. Whether a neuron fires is determined by multiple physical properties, including the current flow through its ion channels, each of which has its own response to temperature change. Two kinds of these, called the transient outward current and the hyperpolarization-activated inward current, oppose each other, and the authors found that each had a high Q_10_.

Turning to computer models of neuronal firing, they demonstrated that not all model neurons were able to adjust to temperature changes while maintaining an appropriate firing pattern. In a model of one of the crab's neurons, they found that temperature change altered the phase of the neuron when the Q_10_ of the membrane currents was set to 1, but the phase was largely maintained when they substituted the experimentally measured Q_10_'s from the two opposing currents.

This is no coincidence, the authors argue. Like all poikilotherms, the crab is subject to strong selective pressure in an environment where temperatures fluctuate, and selection of membrane ensembles that maintain neuronal firing integrity in the face of wide temperature swings is likely to be strongly favored. These exact results may not be found in other cold-blooded creatures, since the precise composition of their neuronal membranes may differ. But since both a crab and a cod must solve the same problem in the cold Atlantic waters, they may be more similar at the cellular level than they appear to be on your plate.

**Tang LS, Goeritz M, Caplan JS, Taylor AL, Fisek M, et al. (2010) Precise Temperature Compensation of Phase in a Rhythmic Motor Pattern. doi:10.1371/journal.pbio.1000469**

